# Characterization of Thermal Expansion Coefficient of 3D Printing Polymeric Materials Using Fiber Bragg Grating Sensors

**DOI:** 10.3390/ma17184668

**Published:** 2024-09-23

**Authors:** Constantina Matsika-Klossa, Nikoleta Chatzidai, Charoula Kousiatza, Dimitrios Karalekas

**Affiliations:** Department of Industrial Management and Technology, University of Piraeus, 18534 Piraeus, Greece; kmatsika@unipi.gr (C.M.-K.); nchatzi@unipi.gr (N.C.); charoula.kousiatza@gmail.com (C.K.)

**Keywords:** coefficient of thermal expansion, glass transition temperature, residual strains, fiber Bragg gratings, raster orientation, Fused Deposition Modeling, thermoplastic materials, carbon fiber-reinforced polymers

## Abstract

This work aims at the determination of the coefficient of thermal expansion (CTE) of parts manufactured through the Fused Deposition Modeling process, employing fiber Bragg grating (FBG) sensors. Pure thermoplastic and composite specimens were built using different commercially available filament materials, including acrylonitrile butadiene styrene, polylactic acid, polyamide, polyether-block-amide (PEBA) and chopped carbon fiber-reinforced polyamide (CF-PA) composite. During the building process, the FBGs were embedded into the middle-plane of the test specimens, featuring 0° and 90° raster printing orientations. The samples were then subjected to thermal loading for measuring the thermally induced strains as a function of applied temperature and, consequently, the test samples’ CTE and glass transition temperature (T_g_) based on the recorded FBG wavelengths. Additionally, the integrated FBGs were used for the characterization of the residual strain magnitudes both at the end of the 3D printing process and at the end of each of the two consecutively applied thermal cycles. The results indicate that, among all tested materials, the CF-PA/0° specimens exhibited the lowest CTE value of 14 × 10^−6^/°C. The PEBA material was proven to have the most isotropic thermal response for both examined raster orientations, 0° and 90°, with CTE values of 117 × 10^−6^/°C and 108 × 10^−6^/°C, respectively, while similar residual strains were also calculated in both printing orientations. It is presented that the followed FBG-based methodology is proven to be an excellent alternative experimental technique for the CTE characterization of materials used in 3D printing.

## 1. Introduction

Additive Manufacturing (AM) is a fast-growing technology which has gained significant attention since its inception in the 1980s, owing to its great advantage of producing structures with intricate geometrical features that are difficult or even impossible to be manufactured via conventional subtractive techniques. Even though the adoption of AM in various industries was initially centered around prototyping purposes, there is nowadays an immensely increasing need for the direct manufacture of functional end-use products via AM methods.

Fused Deposition Modeling (FDM) is one of the most popular and widely spread AM techniques, and is based on the idea of melting extrusion of a polymeric filament and subsequently depositing it in the form of adjacent rasters on a build platform. The rasters’ re-solidification initially leads to the formation of 2D cross sections which stack together in a layer-by-layer manner in order to create the final complex 3D structures. So far, a great variety of pure thermoplastic materials are available to be used in the FDM process. Furthermore, filaments with fiber reinforcement in the polymeric matrix have been developed, especially for high-performance applications, due to the enhanced mechanical and thermal properties that they provide [1,2,3]. The reinforcement can be in the form of discontinuous (chopped) or continuous (long) fibers of carbon, and of glass or aramid material [4,5,6,7]. In general, the most suitable printing material is selected each time, depending on the performance and particular properties required for the final structural component.

The intricate internal architecture of the final FDM structures, composed of partially bonded rasters and voids, governs their overall thermo-mechanical behavior. This internal architecture, leading to anisotropic properties, is strongly dependent on the selected process parameters, such as printing speed, printing orientation, nozzle and build platform temperatures, etc., as well as on the manufacturing process itself, which is featured by the exhibition of non-uniform temperature gradients and recurrently occurred heating and rapidly cooling cycles. Therefore, the final properties of the FDM parts can deviate from the feedstock’s bulk material properties. Furthermore, the aforementioned heating and cooling cycles, exhibited during the deposition process, provoke the development and accumulation of residual stresses and strains in the fabricated components. Apart from their influence on the thermo-mechanical performance of the 3D-printed parts, thermal residual stresses and strains can lead to manufacturing and product quality issues, such as a part’s detachment from the build platform, distortions and warpage phenomena, intra- and/or inter-layer cracking, dimensional inaccuracy or even overall failure of the structure [6,8,9,10,11,12]. Based on all of the above, it is evident that knowledge of the residual strains and/or stresses and thermal and mechanical properties of FDM parts produced by different filament materials is essential in order to enable safe design of structural components and facilitate the widespread adoption of end-use 3D-printed parts.

While extensive research has been undertaken up to now, with a focus on the mechanical properties of parts produced via FDM, the published research data regarding the thermal properties of 3D-printed structures are still scattered. More specifically, among the various thermal properties, the coefficient of thermal expansion (CTE), defined as the change in unit length per degree of temperature change, is a material property of great importance, since it is highly related to thermal deformation, as well as the induced residual stresses and strains in the produced FDM components. Thus, CTE’s characterization for the various different filament materials available in the market with respect to the selected manufacturing process parameters is more than critical.

Based on the available literature, a number of different experimental techniques have been employed for the CTE’s determination of FDM parts fabricated using various thermoplastic or composite feedstock materials. Botean [13] investigated the linear CTE of polylactic acid (PLA), employing an optical method for measuring deformations called Digital Image Correlation (DIC). Baker et al. [14] conducted CTE measurements in both the axial and transverse directions of FDM specimens composed of acrylonitrile butadiene styrene (ABS), polyurethane (PU), polylactic acid PLA and conductive PLA, using a dilatometer. In addition, a differential dilatometer was employed in the research work conducted by Motoc et al. [15] for the examination of the linear CTE of PLA 3D-printed samples with different infill densities. Radulescu et al. [16] proposed a relatively simple device to allow direct observation of spiral polymeric test samples during thermal or negative thermal expansion. The spiral test parts were manufactured by the 3D printing of four different polymeric materials, namely ABS, PLA, PU and polyethylene terephthalate (PET). It was found that the negative thermal expansion had the highest values in the case of the PET material, while the lowest values were obtained in the case of thermoplastic PU. In [17], thermomechanical analysis (TMA) tests were performed in three different measuring directions (axial, transverse and through the thickness) for FDM samples made from ultra-performance materials, namely polyetherimide (PEI) and polyetherketoneketone (PEKK), in order to assess the anisotropy of linear CTE and irreversible thermal strains. Bute et al. [18] also performed TMA tests for the evaluation of the linear CTE and irreversible thermal strains in three directions (X, Y and Z) for various commonly used thermoplastic materials in FDM technology. In terms of CTE’s investigation in composite materials, Faust et al. [19] conducted Dynamic Mechanical Analysis (DMA) tests on specimens made of nylon-based filaments reinforced with discontinuous carbon fibers, as well as of unreinforced nylon-based polymers. The CTE of all considered materials was determined for XY and ZX printing orientations with 0°, ±45° and 90° infill patterns. Based on the obtained results, it was shown that the CTE is dependent on the printing orientation of the part. Additionally, it was concluded that nylons reinforced with discontinuous carbon fiber present more anisotropic CTE properties compared to unreinforced nylon material.

Even though there is very limited research work conducted in this area, an excellent alternative experimental technique for the CTE’s evaluation is presented to be the use of fiber Bragg grating sensors (FBGs). FBGs provide a wide range of advantages, including micro-strain accuracy, non-invasive embedment due to their small size and light weight characteristics, fast response, high sensitivity, signal integrity, long-term stability, immunity against electromagnetic radiation, insensitivity to radio frequency interference and good corrosion resistance [20,21]. Additionally, FBGs can be suitably embedded within a structure, leading to a good interfacial bonding between the sensor and the host material for proper stress/strain transfer [22]. Based on the abovementioned, Economidou et al. [23] investigated the applicability of FBGs for measuring the CTE of FDM parts made of ABS material. The specimens were fabricated with various different raster orientations, namely 0°, 30°, 45°, 60° and 90° in respect to the samples’ long axis. It was shown that the CTEs presented mild variations as a function of raster orientation. Kousiatza et al. In [6], authors performed CTE measurements on 3D-printed continuous fiber-reinforced thermoplastic composites through the embedment of FBGs sensors within the built parts. The composite samples consisted of carbon or glass fiber reinforcement, having various fiber reinforcement orientations, namely ±45°, 0° and 90° or ±45° with respect to the samples’ longitudinal axis, respectively. The results indicated that the calculated CTE values are vigorously affected by the fiber type and orientation. Furthermore, in [24], fiber optic Bragg gratings were used for measuring the CTE values in 3D-printed continuous carbon fiber-reinforced polymeric composites. All of these works clearly demonstrate the FBG sensors’ ability to accurately determine the thermal expansion behavior of pure thermoplastic and composite structures fabricated via the FDM process.

The current study focuses on the CTE’s experimental characterization of FDM parts fabricated from pure thermoplastic and composite materials through the integration of FBG sensors within the test samples’ middle-plane. The specimens were 3D printed using five different commercially available filament materials, namely acrylonitrile butadiene styrene (ABS), polylactic acid (PLA), polyamide (PA), polyether-block-amide (PEBA) and chopped carbon fiber-reinforced polyamide (CF-PA) composite. FBG recordings were derived for the calculation of the developed thermal strains. Then, the CTEs of the built test samples were calculated by taking the slope of the linear segment of the thermal strains vs. temperature graphs, while the specimens’ glass transition temperature (T_g_) was also determined. The effect of the rasters’ deposition orientation on the CTEs’ behavior was identified. Finally, the residual strain magnitudes were investigated via the embedded FBGs, at a post-fabrication state, where the test samples were fully detached from the build platform, as well as at the end of each of the two consecutively applied thermal cycles.

## 2. Materials and Methods

### 2.1. Test Specimens’ Fabrication and Thermal Cycling

**Figure 1:**
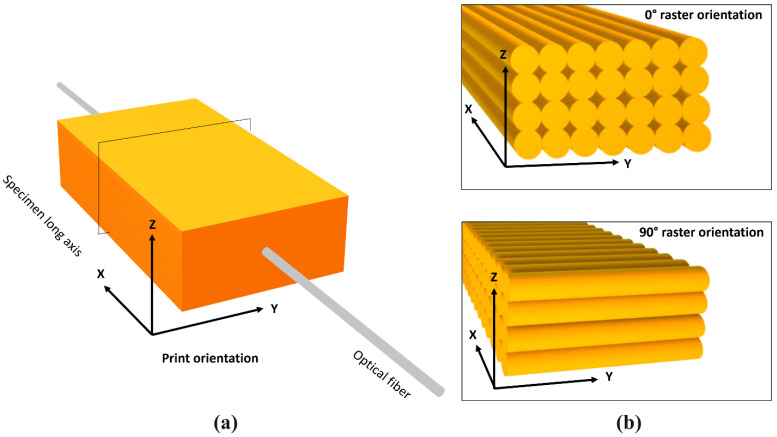
Schematic representation of (**a**) the FBG sensor location within the 3D-printed specimen and (**b**) the raster orientations in respect to the specimens’ long axis.

Five commercially available filament materials were used to fabricate two test specimens per studied material for CTE measurements, namely ABS, PLA, PA, PEBA and CF-PA composite. The test samples with dimensions 8.4 × 40 × 20 mm^3^ were printed using a Flashforge (Flashforge 3D Technology Co., Zhejiang, China) Creator 3 desktop 3D printer. The nozzle and bed temperatures for specimens’ manufacture are given in Table 1. The print head speed was 3000 mm/min. The layer thickness was set at 0.2 mm with 100% infill. Specimens of 42 layers were built with raster orientations of 0° and 90° in respect to the specimens’ long axis, as shown in Figure 1b.

The specimens were subjected to thermal cycling under no mechanical stimulus in an environmental test chamber with a temperature programming system. A K-type thermocouple was placed in the vicinity of each test specimen for recording the temperature close to the specimen area. Heating involved increasing the temperature in a stepwise manner by 1 °C/min for 10 min, starting from around 30 °C up to 120 °C. Upon reaching each temperature plateau, the temperature within the chamber was kept constant for 15 min to achieve thermal equilibrium between the specimen and the surrounding environment before recording the Bragg peak wavelength value. A low heating rate was selected to avoid thermal gradients in the specimens during the heating stage. The materials’ CTE was calculated by taking the slope of the linear segment of the thermal strain ($\varepsilon_{m}^{T}$) vs. temperature (*T*) graphs. At the end of the heating cycle, the specimens were left to cool naturally back to room temperature. The heating–cooling cycle was repeated sequentially twice.

Single FBG sensors (ATGrating Technologies Co., Ltd., Shenzhen, China) were longitudinally embedded in the specimens’ midplane (on top of 21st layer), as presented in Figure 1a. The sensors employed a low attenuation 125 μm diameter standard single mode fiber (SMF28-C). The length of the uncoated inscribed grating was 3 mm and the resulting reflection spectra were in the 1550 nm (±0.5 nm) wavelength range. Spectral measurements were obtained using a FiberSensing FS2100/FS2200 BraggMETER (Hottinger Brüel & Kjær GmbH, Darmstadt, Germany) interrogator equipped with four optical channels. Initial wavelength measurements were performed prior to the printing process at room temperature (23 °C ± 2 °C), as well as at the end of the specimens’ fabrication. Afterwards, wavelength measurements were taken throughout each heating cycle and at the end of each cooling cycle. The wavelength measurements taken at room temperature were used as the reference value (*λ_B_*_0_) for calculating the residual strains at the end of the printing process and the applied thermal cycles.

The materials’ CTE was obtained from the second heating stage, since it is reported by Economidou et al. [23] that the first thermal cycle is expected to lead to unreliable CTE values. This is due to the inherent structural characteristics of and voids’ (porosity) presence in mesostructures fabricated via FDM methods. Additionally, the raster orientation can influence the extent of the grating’s integration within the surrounding material. It is reported that when the applied temperature exceeds the material’s T_g_ temperature, the mesostructure gets rearranged and the voids’ geometry is modified, producing alterations to the sensor–polymer material interface. As a result, any initial fiber–host material bonding imperfections are reduced during the first thermal cycle, leading to enhanced interfacial bonding. It is assumed that only voids close to the grating sensor’s position will play a role in the strain transfer mechanism, whereas the ones located away from the interfacial region and the grating length will not influence the FBG–host material strain transfer.

### 2.2. Fiber Bragg Grating Sensors Working Principles

A Bragg grating is a structure inscribed along a length of an optical fiber, resulting in a periodic variation in the refractive index of this fiber core’s segment. The Bragg grating’s fundamental purpose is the selective reflection of light signals combined in a large reflection, which occurs at a specific wavelength, called the Bragg wavelength, *λ_Β_*_0_, while all other light signals at a different wavelength are transmitted unaltered. The reflected signal is featured by a peak centered on the Bragg wavelength, which is linked to the fiber core’s mean effective refractive index, *n_eff_*, and the grating period, *Λ*_0_, through the Bragg condition: *λ_Β_*_0_ *=* 2*n_eff_Λ*_0_ [25,26,27].

External changes occurring across the grating region, including stress, stain or temperature variations, directly affect both *n_eff_* and *Λ*_0_ parameters, thus leading to a shift in the reflected Bragg wavelength peak. When the embedded FBG sensor into a host material is subjected to a homogenous axial strain *ε_x_* (x is the direction of fiber axis) and/or a uniform temperature change ΔT≠0, and given the assumption that the applied to the fiber transverse strains *ε_z_* and *ε_y_* are related to the axial one, *ε_x_* by *ε_y_ = ε_z_ = −ν_f_ ε_x_*, with *v_f_* being the Poisson’s ratio of the fiber (*v_f_* = 0.16–0.19 for E-glass fiber [28]), then the wavelength shift is described as

(1) $$\frac{\Delta \lambda_{Β}}{\lambda_{Β0}}=\left(1-p_{e}\right)\varepsilon_{axial}+\left(1-p_{e}\right)\left(a_{m}-a_{f}\right)\Delta T+\left(a_{f}+\xi \right)\Delta T$$

where *p_e_* is the effective fiber strain–optic constant (*p_e_* ≈ 0.215 [29]), *ξ* is the thermo-optic coefficient of the fiber (*ξ* ≈ 8.3 × 10^−6^/°C [30]), *α_f_* is the CTE of the fiber core (*α_f_* ≈ 8 × 10^−7^/°C [31]) and *α_m_* is the CTE of the host material. In Equation (1), *ε_axial_* accounts for the solidification-induced residual strains during the printing process and the term (*α_m_* − *α_f_*)*ΔT* for the thermal strains developed due to the mismatch between the CTEs of the optical fiber and the host material.

When recordings are taken at room temperature where *ΔT* = 0, Equation (1) is reduced to the following simple form that can be used to calculate the magnitude of the resulted residual strains at the end of the printing and thermal treatment processes:

(2) $$\frac{\Delta \lambda_{Β}}{\lambda_{Β0}}=\left(1-p_{e}\right)\varepsilon_{residual}.$$

When the embedded FBG sensor into a host material is subjected to a uniform temperature change ΔT≠0, while there is lack of any applied mechanical loading, then Equation (1) is replaced by the following one:

(3) $${\Delta \lambda }_{Β}/\lambda_{Β0}=\left(1-p_{e}\right)\varepsilon^{T}+\left(\alpha_{f}+\xi \right)\Delta T$$

where

(4) $$\varepsilon^{T}=\left(a_{m}-a_{f}\right)\Delta T$$

Combining Equations (3) and (4), while solving for $a_{m}\Delta T$, the thermal strains (*ε_m_^Τ^*) in the host material during the heating cycle are given by

(5) $$\varepsilon_{m}^{T}=\frac{{\Delta \lambda }_{Β}/\lambda_{Β0}+\left(1-p_{e}\right)\alpha_{f}\Delta T-\left(\alpha_{f}+\xi \right)\Delta T}{1-p_{e}}.$$

The wavelength measurements derived by the integrated FBG sensors during the two consecutive heating cycles were used to calculate the thermal strains in the host materials based on Equation (5). The materials’ CTE can be calculated by taking the slope of the linear segment of the thermal strain ($\varepsilon_{m}^{T}$) vs. temperature (*T*) graphs.

## 3. Results and Discussion

**Figure 2:**
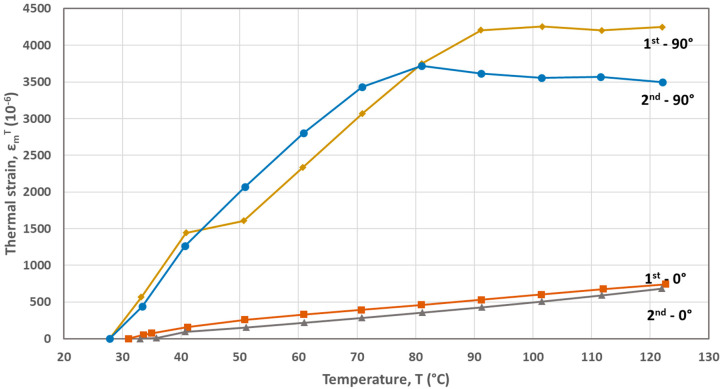
Thermal strains developed during heating cycles for the CF-PA material (0° and 90° raster orientations).

**Figure 3:**
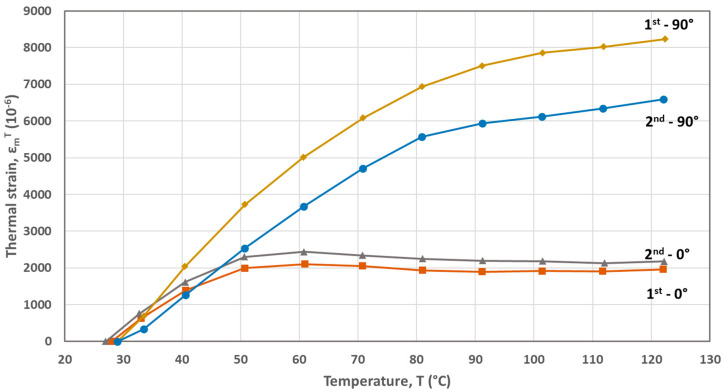
Thermal strains developed during heating cycles for the PA material (0° and 90° raster orientations).

**Figure 4:**
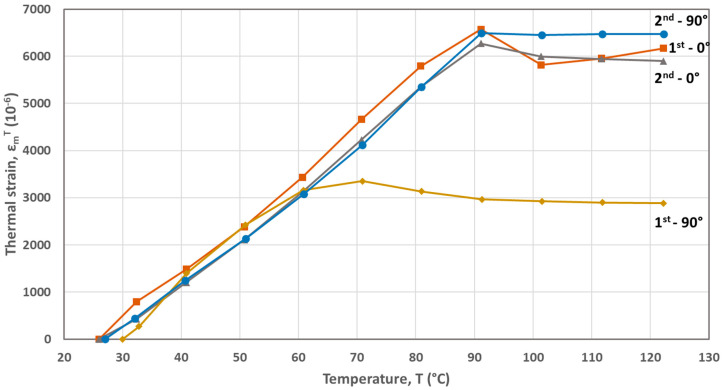
Thermal strains developed during heating cycles for the ABS material (0° and 90° raster orientations).

**Figure 5:**
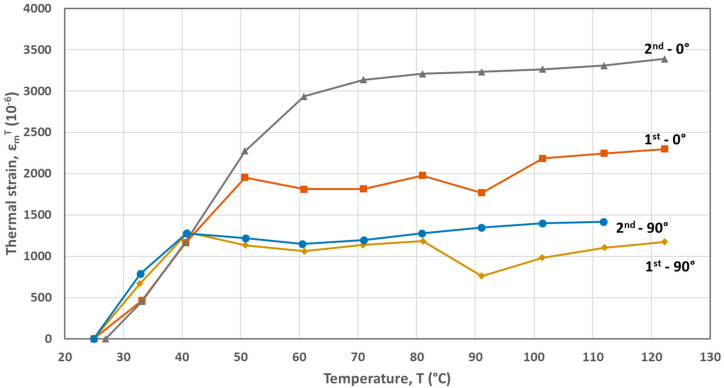
Thermal strains developed during heating cycles for the PLA material (0° and 90° raster orientations).

**Figure 6:**
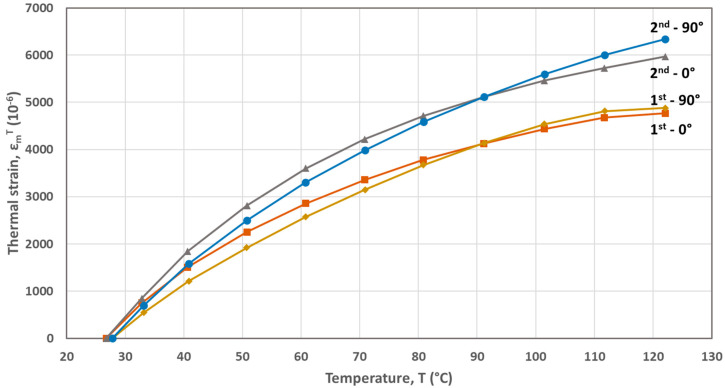
Thermal strains developed during heating cycles for the PEBA material (0° and 90° raster orientations).

The FBG-based calculated thermal strains developed during the first and second heating cycles are plotted as a function of temperature in Figure 2, Figure 3, Figure 4, Figure 5 and Figure 6 for the five materials. As mentioned above, the CTEs were obtained from the second heating cycle, since microstructural changes (e.g., voids filling) are expected to take place during the first one, leading to alterations at the FBG sensor–host material interface resulted at the end of the printing process. The first thermal cycle is reported in [23] to improve the sensor–host material bonding and enhance the strain transfer between the polymer material and the measuring grating of the fiber sensor. From the thermal strain vs. temperature plots presented in Figure 3, Figure 4, Figure 5 and Figure 6 corresponding to PA, ABS, PLA and PEBA, it is seen that during the initial thermal response of the tested materials, in both 0° and 90° raster orientations, similar CTE values can be calculated for both thermal cycles. It is worth mentioning that CTE determination from the recorded FBG wavelength measurements is based on the local in-situ response of the thermally loaded FDM printed materials, eliminating thus the effect of any fabrication non-uniformities (e.g., non-uniform density due to gaps of different sizes) that can affect the values of the calculated CTE [16].

The overall thermal expansion behavior of the CF-PA material is presented to be quite similar for both sequential heating cycles corresponding to the 0° and 90° raster orientations, respectively. The same behavior is also observed for PA and PEBA in both the 0° and 90° raster orientations, as well as for ABS/0° and PLA/90° materials. It is seen from Figure 2 that the thermal behavior of the CF-PA/0° orientation is stable, leading to the same CTE value of α = 14 × 10^−6^/°C throughout both applied thermal cycles. This low magnitude designates the crucial role that the short carbon fibers, aligned along the 0° printing direction, play in the thermomechanical behavior of the printed specimen. On the other hand, the CF-PA/90° thermal expansion is influenced by the expansion of the PA matrix.

The CTEs calculated from the graphs of the second heating cycle are presented in Table 2. It is evident from Figure 2, Figure 3, Figure 4, Figure 5 and Figure 6 that along the thermal strain vs. temperature plots, different linear sections can be identified to calculate the corresponding CTE values. In Table 2, only the CTE values of the tested materials at a 25 °C to around 50 °C temperature range are presented along with ones reported in other research works. It is seen that the FBG-based CTE values are comparable to the ones reported in the literature and that the semi-crystalline polymers such as PA, PLA and PEBA have a higher CTE than amorphous ones like ABS.

It is important to mention that the CTE values of the printed materials reported in the present study would be different from those of the corresponding bulk materials. However, for the ABS, PLA and PA printed materials, the FBG-based calculated CTEs are comparable to the ones reported in other research works, where different experimental methods were used, such as thermomechanical analysis in [18] or the dilatometer in [14].

For the unreinforced materials PA, PLA and ABS, the peak of their curves designate the point at which the materials soften entering in a viscous state. This softening temperature could be used as an approximation of the materials T_g_. The T_g_ values for these materials are also presented in Table 2. On the other hand, no softening point is determined for the CF-PA/0° and PEBA (0° and 90° orientations) materials which exhibited identical thermal response for both thermal cycles.

**Figure 7:**
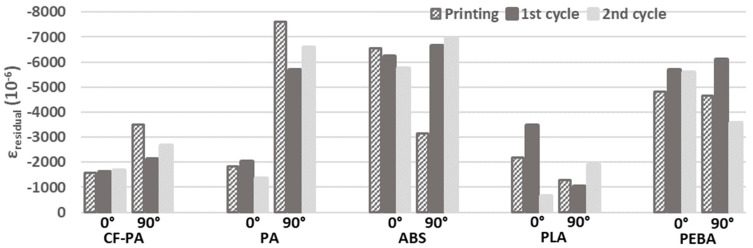
Resulted residual strains at the end of the printing process and at the end of the two thermal cycles for the studied materials and raster orientations.

The resulted residual strains at the end of the printing process and at the end of the two consecutively applied thermal cycles are presented in Table 3 and in Figure 7. All measurements were taken at room temperature with the test specimens resting in an unconstrained manner on the printing bed and oven platform, correspondingly.

At the end of the 3D printing process, it is observed that the magnitude of the calculated residual strains is considerable. The anisotropic character of the printed structures resulted in noticeable differences in the calculated strains for all materials, except for PEBA, when the rasters’ deposition orientation was parallel and perpendicular to the measuring grating of the optical fiber.

Regarding the CF-PA and PA materials, higher strains were developed in the 90° compared to 0° printing direction in all cases. On the other hand, the 0° building orientation led to higher strains for the PLA material. As far as the PEBA is concerned, similar residual strains were calculated in both printing orientations at the end of the printing process as well as at the end of the first thermal cycle, demonstrating a more isotropic structural behavior. Comparable strain values were also observed for the ABS material at the end of the first and second thermal cycling, respectively, due to internal gaps filling, which led to a more isotropic mesostructure. The lowest residual strains were obtained for the CF-PA/0°, PA/0°, and PLA/0° and PLA/90° materials.

## 4. Conclusions

The purpose of this study was to investigate the thermal expansion characteristics of five common thermoplastic materials using an embedded FBG sensor in 3D-printed samples. The recorded FBG wavelength changes induced with temperature increase in a preselected thermal cycle were used to calculate the resulted thermal strains during heating. These strains, when plotted versus temperature, led to the determination of the materials’ CTE from the initial linear section of the plot. Additionally, the embedded FBG sensor was used to evaluate the resulted residual strains in the test samples at the end of the printing process and at the ends of the consecutively applied thermal cycles.

The main results of the study were as follows:

1. The locally calculated CTEs and T_g_ values, based on the FBG sensor response to temperature increase, are comparable to the CTEs reported in literature, determined using other measuring methods, such as TMA or dilatometry.
2. For most of the tested materials, their thermal response was similar in both consecutively applied thermal cycles.
3. The CF-PA/0° specimens exhibited the lowest CTE value of 14 × 10^−6^/°C, as a result of the presence of the short carbon fibers aligned in the 0° printing orientation.
4. The PEBA material was proven to have the most isotropic thermal response for both examined raster orientations of 0° and 90°, with CTE values of 117 × 10^−6^/°C and 108 × 10^−6^/°C, respectively. This isotropic behavior is also evident from the similar residual strains calculated in both printing orientations at the end of the printing process as well as at the end of the first thermal cycle.
5. The sensor-based calculated residual strains were of considerable magnitude from measurements taken at room temperature at the end of the printing process and at the ends of the applied thermal cycles.
6. Considering that the present work has validated the methodology of measuring CTE using FBG sensors on a wide range of 3D-printed materials, a future research work could use FBG sensors for mechanical characterization of the studied materials.

## Figures and Tables

**Table 1:** Materials’ printing temperatures.

Material	Manufacturer	Selected Extruder Temperature, (°C)	Selected Bed Temperature, (°C)
CF-PA	Addigy Covestro—ID1030 CF10 (Covestro AG, Leverkusen, Germany)	285	100
PA	Taulman—Bridge Nylon (Braskem SA, São Paulo, Brazil)	255	65
ABS	eSUN—Gray (Shenzhen Esun Industrial Co., Shenzhen, China)	240	100
PLA	PM—White (Plasty Mladeč, Haňovice, Czech Republic)	210	65
PEBA	Flexfill—PEBA 90A (Fillamentum Manufacturing Czech s.r.o., Hulin, Czech Republic)	245	100

**Table 2:** Coefficients of thermal expansion and T_g_ for all studied materials.

Material	Raster Orientation	Temperature Range, (°C)	CTE, (10^−6^/°C)	Literature CTE, (10^−6^/°C)	T_g_ (°C)	Literature T_g_, (°C)
CF-PA	0°	25–40	14 ± 1	22 [19]	**-**	**-**
	90°	25–60	86 ± 3	95 [19]	82 ± 2	**-**
PA	0°	25–40	118 ± 5	126 [18]	52 ± 2	50–55 [32]
	90°	25–50	119 ± 5	120 [18]	82 ± 2	**-**
ABS	0°	25–50	86 ± 3	90 [18], 102 [23]	92 ± 2	107 [33]
	90°	25–50	89 ± 3	93 [18], 115 [23]	92 ± 2	**-**
PLA	0°	25–50	96 ± 5	100 [18]	62 ± 2	57 [33]
	90°	25–30	99 ± 5	102 [18]	42 ± 2	**-**
PEBA	0°	25–50	117 ± 5	**-**	**-**	**-**
	90°	25–40	108 ± 5	**-**	**-**	**-**

**Table 3:** FBG-based measured residual strains.

Material	Raster Orientation	Measured Residual Strains, ε (10^−6^)		
		End of Printing	End of 1st Thermal Cycle	End of 2nd Thermal Cycle
CF-PA	0°	−1571	−1607	−1663
	90°	−3514	−2114	−2681
PA	0°	−1842	−2040	−1361
	90°	−7615	−5678	−6588
ABS	0°	−6565	−6231	−5753
	90°	−3156	−6643	−6944
PLA	0°	−2188	−3465	−644
	90°	−1273	−1034	−1882
PEBA	0°	−4807	−5671	−5579
	90°	−4664	−6099	−3554

## Data Availability

The data presented in this research are available on request from the corresponding authors.

## References

[1] Quintana J.L.C., Slattery L., Pinkham J., Keaton J., Lopez-Anido R.A., Sharp K. (2022). Effects of Fiber Orientation on the Coefficient of Thermal Expansion of Fiber-Filled Polymer Systems in Large Format Polymer Extrusion-Based Additive Manufacturing. Materials.

[2] Love L.J., Kunc V., Rios O., Duty C.E., Elliott A.M., Post B.K., Smith R.J., Blue C.A. (2014). The Importance of Carbon Fiber to Polymer Additive Manufacturing. J. Mater. Res..

[3] Ning F., Cong W., Qiu J., Wei J., Wang S. (2015). Additive Manufacturing of Carbon Fiber Reinforced Thermoplastic Composites Using Fused Deposition Modeling. Compos. Part B Eng..

[4] Polyzos E., Van Hemelrijck D., Pyl L. (2024). Modeling of the Coefficient of Thermal Expansion of 3D-Printed Composites. Int. J. Mech. Sci..

[5] Iragi M., Pascual-González C., Esnaola A., Lopes C., Aretxabaleta L. (2019). Ply and Interlaminar Behaviours of 3D Printed Continuous Carbon Fibre-Reinforced Thermoplastic Laminates; Effects of Processing Conditions and Microstructure. Addit. Manuf..

[6] Kousiatza C., Tzetzis D., Karalekas D. (2019). In-Situ Characterization of 3D Printed Continuous Fiber Reinforced Composites: A Methodological Study Using Fiber Bragg Grating Sensors. Compos. Sci. Technol..

[7] Melenka G.W., Cheung B.K.O., Schofield J.S., Dawson M.R., Carey J.P. (2016). Evaluation and Prediction of the Tensile Properties of Continuous Fiber-Reinforced 3D Printed Structures. Compos. Struct..

[8] Zhang W., Wu A.S., Sun J., Quan Z., Gu B., Sun B., Cotton C., Heider D., Chou T.-W. (2017). Characterization of Residual Stress and Deformation in Additively Manufactured ABS Polymer and Composite Specimens. Compos. Sci. Technol..

[9] Sunny S., Chen H., Malik A., Lu H. (2021). Influence of Residual Stress and Fluid–Structure Interaction on the Impact Behavior of Fused Filament Fabrication Components. Addit. Manuf..

[10] Spoerk M., Sapkota J., Weingrill G., Fischinger T., Arbeiter F., Holzer C. (2017). Shrinkage and Warpage Optimization of Expanded-Perlite-Filled Polypropylene Composites in Extrusion-Based Additive Manufacturing. Macromol. Mater. Eng..

[11] Fitzharris E.R., Watanabe N., Rosen D.W., Shofner M.L. (2018). Effects of Material Properties on Warpage in Fused Deposition Modeling Parts. Int. J. Adv. Manuf. Technol..

[12] Chatzidai N., Karalekas D. (2019). Experimental and Numerical Study on the Influence of Critical 3D Printing Processing Parameters. Frat. Ed Integrita Strutt. Struct. Integr..

[13] Botean A.-I. Thermal Expansion Coefficient Determination of Polylactic Acid Using Digital Image Correlation. Proceedings of the EENVIRO 2017 Workshop—Advances in Heat and Transfer in Built Environment.

[14] Baker A.M., McCoy J., Majumdar B.S., Rumley-Ouellette B., Wahry J., Marchi A.N., Bernardin J.D., Spernjak D. Measurement and Modelling of Thermal and Mechanical Anisotropy of Parts Additively Manufactured Using Fused Deposition Modelling (FDM). Structural Health Monitoring 2017: Real-Time Material State Awareness and Data-Driven Safety Assurance. Proceedings of the 11th International Workshop on Structural Health Monitoring, IWSHM.

[15] Motoc D.L., Draghicescu H.T., Florea D., Preda I., Ispas N. (2021). Insights into Mechanical and Thermal Properties of Additively Manufactured PLA Samples Triggered by Automotive Industry Demands. Mater. Plast..

[16] Rădulescu B., Mihalache A.M., Hrițuc A., Rădulescu M., Slătineanu L., Munteanu A., Dodun O., Nagîț G. (2022). Thermal Expansion of Plastics Used for 3D Printing. Polymers.

[17] Glaskova-Kuzmina T., Dejus D., Jātnieks J., Vīndedze E., Bute I., Sevcenko J., Aniskevich A., Stankevich S., Boobani B. (2024). The Tensile, Thermal and Flame-Retardant Properties of Polyetherimide and Polyetherketoneketone Processed via Fused Filament Ffabrication. Polymers.

[18] Bute I., Tarasovs S., Vidinejevs S., Vevere L., Sevcenko J., Aniskevich A. (2023). Thermal Properties of 3D Printed Products from the Most Common Polymers. Int. J. Adv. Manuf. Technol..

[19] Faust J.L., Kelly P.G., Jones B.D., Roy-Mayhew J.D. (2021). Effects of Coefficient of Thermal Expansion and Moisture Absorption on the Dimensional Accuracy of Carbon-Reinforced 3D Printed Parts. Polymers.

[20] Kousiatza C., Karalekas D. (2016). In-Situ Monitoring of Strain and Temperature Distributions during Fused Deposition Modeling Process. Mater. Des..

[21] Kinet D., Mégret P., Goossen K.W., Qiu L., Heider D., Caucheteur C. (2014). Fiber Bragg Grating Sensors toward Structural Health Monitoring in Composite Materials: Challenges and Solutions. Sensors.

[22] Quattrocchi A., Montanini R. (2023). Development and Verification of Self-Sensing Structures Printed in Additive Manufacturing: A Preliminary study. Acta IMEKO.

[23] Economidou S.N., Karalekas D. (2016). Optical Sensor-Based Measurements of Thermal Expansion Coefficient in Additive Manufacturing. Polym. Test..

[24] Orlowska-Galezia A., Graczykowski C., Pawlowski P., Rimašauskienė R., Rimašauskas M., Kuncius T., Majewska K., Mieloszyk M. (2024). Characterization of Thermal Expansion in Additively Manufactured Continuous Carbon Fibre Reinforced Polymer Composites Using Fibre Bragg Grating Sensors. Measurement.

[25] Kashyap R. (2009). Fiber Bragg Gratings.

[26] Measures R., Abrate S. (2002). Structural Monitoring with Fiber Optic Technology. Appl. Mech. Rev..

[27] Othonos A., Kalli K. (1999). Fiber Bragg Gratings: Fundamentals and Applications in Telecommunications and Sensing.

[28] Colpo F., Humbert L., Botsis J. (2007). Characterisation of Residual Stresses in a Single Fibre Composite with FBG Sensor. Compos. Sci. Technol..

[29] Giaccari P., Dunkel G.R., Humbert L., Botsis J., Limberger H.G., Salathé R.P. (2005). On a Direct Determination of Non-Uniform Internal Strain Fields Using Fibre Bragg Gratings. Smart Mater. Struct..

[30] Shibata N., Shibata S., Edahiro T. (1981). Refractive Index Dispersion of Lightguide Glasses at High Temperature. Electron. Lett..

[31] Lai M., Friedrich K., Botsis J., Burkhart T. (2010). Evaluation of Residual Strains in Epoxy with Different Nano/Micro-Fillers Using Embedded Fiber Bragg Grating Sensor. Compos. Sci. Technol..

[32] Krause B., Kroschwald L., Pötschke P. (2019). The Influence of the Blend Ratio in PA6/PA66/MWCNT Blend Composites on the Electrical and Thermal Properties. Polymers.

[33] Sirjani E., Cragg P.J., Dymond M.K. (2019). Glass Transition Temperatures, Melting Temperatures, Water Contact Angles and Dimensional Precision of Simple Fused Deposition Model 3D Prints and 3D Printed Channels Constructed from a Range of Commercially Available Filaments. Chem. Data Collect..

